# O.4.5-9 Moderate-to-vigorous physical activity assessed by tri-axial Accelerometers and its association with the risk of obesity in 6-10 years-old children

**DOI:** 10.1093/eurpub/ckad133.217

**Published:** 2023-09-11

**Authors:** Aristides M Machado-Rodrigues, Daniela Rodrigues, Augusta Gama, Helena Nogueira, Maria-Raquel Silva, Luís P Mascarenhas, Cristina Padez

**Affiliations:** Research Centre for Anthropology and Health, University of Coimbra, Portugal; Faculty of Sport Sciences and Physical Education, Coimbra, Portugal; Research Centre for Anthropology and Health, University of Coimbra, Portugal; Research Centre for Anthropology and Health, University of Coimbra, Portugal; Research Centre for Anthropology and Health, University of Coimbra, Portugal; Research Centre for Anthropology and Health, University of Coimbra, Portugal; Department of Nutrition, Fernando Pessoa University, Porto, Portugal; Comprehensive Health Research Centre-Group of Sleep, University of Lisbon, Portugal; UniCentro, Department of Physical Education, Midwestern Paraná State University, Brazil; rodriguesari@hotmail.com; Research Centre for Anthropology and Health, University of Coimbra, Portugal

## Abstract

**Purpose:**

There is a worldwide agreement that children should participate in at least 60 min of moderate-to-vigorous physical activity (MVPA) daily to achieve substantial health benefits. Furthermore, using triaxial-accelerometers has become more accurate practice in the study of childhood obesity. The present study aimed: i) to compare PA intensity levels and Body Mass Index (BMI) of boys and girls, and assess MVPA recommendation compliance of active children; ii) to examine associations between the risk of overweight and the MVPA in children.

**Methods:**

The sample of 395 children (198 girls) aged 6-10 years was assessed. Height and weight were measured, and BMI was calculated subsequently. A tri-axial accelerometer was used to obtain seven consecutive days of MVPA, as well as the weekly time being sedentary. Logistic regression analysis was used to analyse the above-mentioned relationship between MVPA and the risk of obesity, controlling for potential confounders.

**Results:**

About 47% of boys were active on week days by achieve the mean value of 60 min/day of MVPA; corresponding percentage for the weekend was just 32%. Among girls, only 22% were active on week days and 29% at the weekend. MVPA was not significantly associated with the risk of being overweight nor in female neither in male children. The final regression model revealed that girls of mothers with high educational level were less likely to be classified as overweight girls.

**Conclusions:**

Findings of the present study revealed no significantly relation between MVPA and obesity risk among Portuguese children. Future research should extend similar design to other lifestyle features of children to clarify potential predictors of being overweight at early ages.

**Support/Funding Source:**

Fundação para a Ciência e a Tecnologia [PEst-OE/SADG/UI0283/2013].

